# Sustainable Materials Based on Geopolymer–Polyvinyl Acetate Composites for Art and Design Applications

**DOI:** 10.3390/polym14245461

**Published:** 2022-12-13

**Authors:** Laura Ricciotti, Alessio Occhicone, Stefania Manzi, Andrea Saccani, Claudio Ferone, Oreste Tarallo, Giuseppina Roviello

**Affiliations:** 1 Department of Architecture and Industrial Design, University of Campania, Luigi Vanvitelli, 81031 Aversa, Italy; 2 Department of Engineering, University of Naples Parthenope, INSTM Research Group Napoli Parthenope, 80143 Naples, Italy; 3 Department of Civil, Chemical, Environmental and Material Engineering, University of Bologna, 40131 Bologna, Italy; 4 Department of Chemical Science, University of Naples Federico II, 80126 Naples, Italy

**Keywords:** geopolymer, composites, PVAc, green materials, art and design, cultural heritage

## Abstract

The recent introduction of the Next Generation EU packages on the circular economy and the Italian Ecological Transition Plan has further boosted the research of effective routes to design materials with low energy and low environmental impact, in all areas of research, including art and design and cultural heritage. In this work, we describe for the first time the preparation and characterization of a new sustainable adhesive material to be used in the art and design sector, consisting of a geopolymer-based composite with polyvinyl acetate (PVAc), both considered more environmentally acceptable than the analogous inorganic or polymeric materials currently used in this sector. The key idea has been the development of organic–inorganic composites by reacting low molecular weight polymers with the geopolymer precursor to obtain a material with reduced brittleness and enhanced adhesion with common substrates. Structural, morphological, and mechanical studies pointed out the consistent microstructure of the composite materials if compared to the neat geopolymer, showing lower density (up to 15%), improved flexural strength (up to 30%), similar water absorption and a relevant toughening effect (up to 40%). Moreover, the easy pourability in complex shapes and the excellent adhesion of these materials to common substrates suggest their use as materials for restoration, rehabilitation of monuments, and decorative and architectural intervention. The organic–inorganic nature of these new materials also makes them easily recognizable from the support on which they are used, favoring, in line with the dictates of good restoration practices, their possible complete removal. For all these reasons, these new materials could represent promising candidates to overcome the limits related to the creative industry for what concerns the selection of environmentally friendly materials to meet design requirements with low environmental impacts.

## 1. Introduction

Modern society regards the preservation and restoration of cultural heritage as a duty to future generations. This is due to the deterioration and continuous damage of artworks and monuments caused by air pollution in industrialized areas [1]. Because of these issues, most archaeological finds, historic buildings, and monuments exhibit conservation problems that require primary restoration works. In current practice, restoration processes are based on the careful analysis of the material artworks in order to design repair and restoration materials with characteristics as similar as possible to those of historical artefacts [2,3]. Compatible materials are chosen not only based on similar aesthetic characteristics but with similar chemical–physical and mechanical properties.

Recently, new materials have been developed as protective coatings for various substrates, such as structural consolidants and protective coatings for ceramics, wood, steel, concrete walls, textiles, and so on [4,5]. Moreover, these materials can be used in architectural engineering and modern and sustainable construction as well as for the restoration of historical and cultural assets [6]. In fact, advanced materials used in the field of art and design and modern architecture have to possess several technological features, such as adequate ductility and penetration capacity, low creeping suitable mechanical properties [7], proper chemical composition, long durability, and flexibility of shape and color. Moreover, low environmental impacts and energy consumption and optimization of the amount of the material to use (minimizing waste) are also required. For these reasons, nowadays, academic research is increasingly focused on developing materials that, in addition to possessing those important technological characteristics, can exhibit remarkable aesthetic qualities and be easily adapted for applications in the fields of art and design and cultural heritage.

In this context, geopolymer-based materials have attracted much attention due to their chemical–physical, morphological, and mechanical properties: chemical and fire resistance, low shrinkage, freeze–thaw, thermal stability, long-term durability, and recyclability [8]. Geopolymers are aluminosilicate-based amorphous materials obtained from the alkaline activation of natural or waste materials such as metallurgical, industrial, urban, and agricultural wastes [9]. The aluminosilicate precursor powder reacts with an activating alkaline solution, consisting of sodium and/or potassium hydroxides and silicates, at a temperature below 100 °C, and it produces a ceramic amorphous matrix. 

These materials are nowadays considered for their potential use in many applications and industrial fields [10,11,12], such as civil engineering, cements and concretes, automotive and aerospace industries, retrofit of buildings, waste management [13], matrices for hazardous waste stabilization, tooling and moldings, fireproof barriers, [14,15] and, as already mentioned, in art and decoration [16,17]. In terms of applications as cement and concrete materials, they can reduce energy consumption during production, emission of greenhouse gases, and environmental impacts [18,19,20,21].

During the last decade, geopolymer-based hybrid or composite materials have been also studied, mainly in order to overcome their brittleness, one of the main issues of the mechanical properties of these materials. The organic phase can be added to the geopolymer paste in a solid form such as powder, natural, and synthetic fibers [22], as well as particles [23,24], or in a liquid form. Recently, Gupta et al. have described an inorganic–organic (Si-O-Al) hybrid geopolymer via the solid state method [25]. In this context, the authors have developed a simple and effective strategy to obtain stable composite [26,27] and hybrid [28] geopolymer materials, even with large amounts of the organic component (up to 20%wt) and without the addition of compatibilizing agents. These systems were synthetized by using a co-reticulation reaction that occurs between the organic phase and the geopolymer. It has been reported that the presence of the organic phase generates a change in the viscosity of the slurry, resulting in a mixture characterized by a good thixotropicity while preserving a good workability, which is useful to mold into different geometries and apply to vertical surfaces [26]. Moreover, these new materials have demonstrated superior mechanical properties compared to neat geopolymer and, in particular, are less brittle and demonstrate an increased compressive strength [29].

To date, composite materials (characterized by spherical domains of micrometric size well dispersed into the geopolymer matrix) have been obtained by mixing epoxy or melamine resins precursors in the geopolymeric mixture, while hybrid materials have been produced by the addition of polydimethylsiloxane (PDMS) oligomers to the geopolymer slurry [29]. In this last case, microstructural analyses revealed a structural homogeneity up to the nanometric level, suggesting that, due to the chemical similarity of the components, strong interactions between polydimethylsiloxane oligomers and geopolymeric units can be achieved. The formation of such a hybrid phase turned out to significantly improve the mechanical and thermal performances compared to the neat geopolymers [29,30].

In this framework, in order to contribute to the development of new materials for applications in the art and design sector, we have prepared new composite geopolymeric materials containing organic polymers with good adhesive properties aiming at improving the mechanical strength of the geopolymer matrix and, at the same time, preserving—or even increasing—its chemical compatibility with different types of substrates. To this aim, the attention has been focused on polyvinyl acetate (PVAc), a polymer characterized by good adhesion to various substrates (such as metals, plastics, and wood) and widely used as a general building adhesive [31,32]. The interest towards this polymer was also boosted by the fact that, in agreement with the current guidelines for the development of an adhesive technology that replaces organic solvent-based adhesives with water-based ones [33], PVAc is a water-based adhesive, and for this reason, it is considered more environmentally acceptable compared to organic–solvent-based adhesives. As a final remark, it is worth pointing out that, thanks to its polar nature, PVAc could be in principle capable of potentially establishing strong chemical interactions with the geopolymer phase, such as hydrogen bonds, thus allowing the obtainment of composite materials characterized by a strong interaction between the organic and inorganic phases.

In this work, the synthesis of new geopolymer-based composites containing up to 10% by weight of polyvinyl acetate and their chemical–physical, morphological, and mechanical properties have been investigated. Such properties have been compared with those of geopolymer–polydimethylsiloxane hybrid materials obtained by a similar experimental procedure, which, to the best of our knowledge, is a class of geopolymer-based materials demonstrating significantly improved performances in neat geopolymers [29]. This study demonstrates that these new geopolymer-based composites can be used in the field of decorative industry and cultural heritage, due to their technological properties such as easy workability, good thixotropocity, easiness of shaping and molding, and suitable mechanical properties. Moreover, since they exhibit an excellent adhesion to different kinds of substrates (such as ceramic, tuff, traditional concrete, marble and earthenware), their application in restoration, conservation, sculptures, and building revetments may be envisaged. 

Particularly, these geopolymer composites materials meet many of the requirements of restoration materials: they bond stably to different substrates, are easily removable (this is the reversibility of intervention, which is a property difficult to find among commonly used materials), has low density (as a ceramic material), good mechanical properties, and easy workability.

## 2. Materials and Methods

### 2.1. Materials 

The metakaolin (BASF MetaMax^®^) used in this work is a high-purity white mineral aluminosilicate precursor that meets all the specifications of ASTM C-618 Class N pozzolans and was kindly provided by Neuvendis s.p.a. (Milan, Italy). The sodium silicate solution was supplied by Prochin Italia S.r.l (Caserta, Italy). The chemical composition of the metakaolin and sodium silicate solution is shown in Table 1. Sodium hydroxide with reagent grade, polyvinyl acetate (PVAc) and polydimethylsiloxane (PDMS) oligomers were supplied by Sigma-Aldrich (Milan, Italy).

### 2.2. Samples’ Preparation

#### 2.2.1. Neat Geopolymer (MK)

The reaction of alkaline activation of the metakaolin (aluminosilicate raw material) with an alkaline activating solution allows one to obtain a geopolymer. Sodium hydroxide (sigma-Aldrich 98%) in pellets was added to sodium silicate solution under constant mechanical stirring. The prepared solution was then allowed to equilibrate at room temperature for 24 h. The composition of the solution can be expressed as Na_2_O 1.55 SiO_2_ 12.14 H_2_O in molar ratio [17,29]. Subsequently, the metakaolin was added into the activating solution with a 1.4:1 by weight in a liquid-to-solid ratio and mechanically mixed at 850 rpm for 10 min [17]. 

The composition of the whole geopolymer system (as determined by EDS analysis on the hardened samples) can be expressed as Al_2_O_3_ 3.48 SiO_2_ 1.0 Na_2_O 12.14 H_2_O in a molar ratio, referring to a complete geopolymerization process. The neat geopolymer sample was indicated as MK.

#### 2.2.2. Geopolymer Composites’ (MK-PVAc-5; MK-PVAc-10; MK-PDMS-5 and MK-PDMS-10) Preparation

The geopolymer-based composites and hybrids were obtained by adding different percentages by weight of liquid vinyl acetate and dimethylsiloxane oligomers to the freshly prepared geopolymer suspension and are quickly incorporated by controlled mechanical mixing (5 min at 800 rpm). The mixture is easily workable for several hours (the complete crosslinking and hardening take place in about 5–7 h at room temperature [16,29]). The samples are hereafter indicated as MK-PVAc-5 and MK-PVAc-10 (in which polyvinyl acetate was 5% and 10% in weight, respectively); MK-PDMS-5 and MK-PDMS-10 (in which the content of polydimethylsiloxane oligomers was 5% and 10% in weight, respectively).

#### 2.2.3. Curing Treatments 

The prepared specimens were poured in cubic molds (size 50 mm × 50 mm × 50 mm and 40 mm × 40 mm × 160 mm) and cured at 60 °C for 24 h in 95% of the relative humidity conditions. Subsequently, the specimens were kept in >95% relative humidity conditions at room temperature for a further 6 days and then for a further 3 weeks in air.

The mix design of the samples is reported in Table 2.

### 2.3. Sample Characterization

#### 2.3.1. Thermogravimetric Analysis

Thermogravimetric analyses (TGA) were made using a TA Instrument SDT2960 simultaneous DSC-TGA (TA Instrument, New Castle, DE, USA). Heating rate was fixed to 10 °C/min, and ≈10 mg of the powdered sample was used.

#### 2.3.2. X-ray Diffraction Characterization

X-ray diffraction patterns were collected at room temperature with Miniflex 600 automatic Rigaku powder diffractometer, operating in the θ/2θ Bragg-Brentano geometry. Phase recognition was carried out by using the Rigaku PDXL2 software and PDF-2-2022 (International Centre for Diffraction Data^®^) database.

#### 2.3.3. Morphological Characterization

Scanning Electron Microscopy (SEM) analysis was carried out using a Nova Nanosem 450 (FEI, Austin, TX, USA) on the freshly obtained fracture surfaces of the samples, metallized with Pd/Au. The acceleration potential used is between 5 and 10 kV.

#### 2.3.4. Physical and Mechanical Properties

As for physical properties, the bulk density (D) and water absorption (WA) of the samples was determined from the half prisms obtained from flexural tests at the age of 28 days. Two specimens of about 40 mm × 40 mm × 20 mm were obtained for each (sample) series. Bulk density was determined by dividing the dry mass by the geometrical volume. Dry mass was obtained after 3 days in an oven (G-Therm 035, F.lli Galli G. & P., Milan, Italy) at 55 °C until it reached a constant mass. Water absorption was obtained by soaking the dried specimens in deionized water at room temperature and atmospheric pressure for 48 h until a constant mass was obtained. WA was calculated as the difference between wet and dry mass divided by the dry mass value. A Kern EG hydrostatic analytical balance (Kern & Sohn GmbH, Balingen, Germany) was used.

As for mechanical properties, the dynamic modulus of elasticity (E), three-point flexural strength (σ_f_), and compressive strength (σ_c_) were determined on 40 mm × 40 mm × 160 mm prisms after 28 days of curing. It was determined using a commercial ultrasonic testing instrument (C369N, Matest, Bergamo, Italy), comprised of a pulse generator and two transducers (55 kHz) that were positioned at the two ends of the 160 mm-long prisms. The dynamic modulus of elasticity is reported as the average of three measurements. Flexural and compressive strengths were performed according to EN 196–1 at a speed rate of 50 mm/min with a 100 kN Amsler Wolpert testing machine (Ludwighafen, Germany) at 22 ± 1 °C and 65 ± 5 RH%. Flexural and compressive strengths herein reported are the average of three and five measurements, respectively.

## 3. Results and Discussion

### 3.1. Thermogravimetric Analysis

Thermogravimetric analyses were performed on the neat geopolymer MK and geopolymer composites MK-PDMS-10 and MK-PVAc-10 to investigate their thermal behavior.

**Figure 1:**
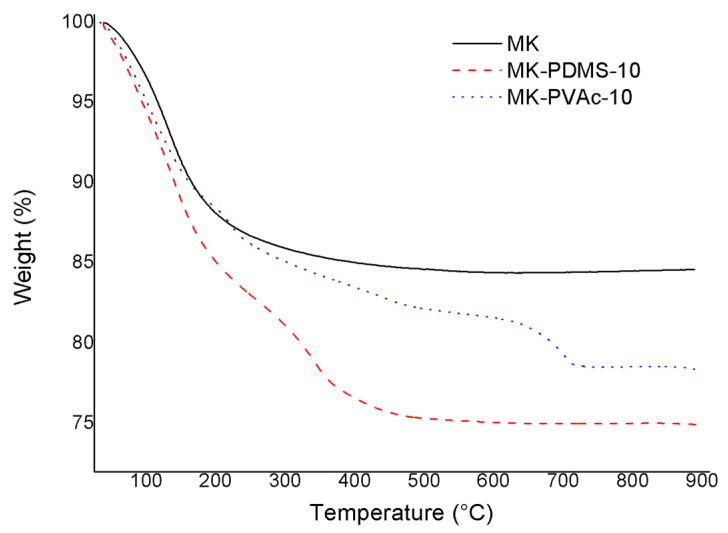
TGA curves of MK (black continuous line), MK-PDMS-10 (red dashed line), and MK- PVAc-10 (blue dotted line) geopolymer samples.

The corresponding curves are shown in Figure 1.

The MK geopolymer sample weight loss starts at ≈30 °C with the inflexion point at a temperature of around 120–130 °C, and a loss that is completed at ≈300 °C. This behavior has been attributed to the removal of water molecules absorbed (up to ≈100 °C) or differently linked (up to ≈200 °C) within the pores [10]. For structural and bound water in nanopores, this weight loss can occur at higher temperatures compared to the silicate matrix [34].

As for the MK-PVAc-10 and MK-PDMS-10 composites, a first step, corresponding to a weight loss of ≈8%, occurs up to ≈150 °C, while a second step is observed (up to ≈500 °C for MK-PDMS-10 and ≈720 °C for MK-PVAc-10) and corresponds to a further weight loss equal to ≈10%. From the comparison of the thermogravimetric profile of the neat geopolymer MK and the composite ones (see Figure 1), the first degradation step (up to ≈250–300 °C) is likely associated to the loss of water of the geopolymeric phase, while the second one (ending at ≈450 °C in the case of MK-PDMS and ≈720 °C in the case of MK-PVAc) likely corresponds to the degradation of the dispersed organic phase. The combustion residual at 800 °C is about 78% for MK-PVAc-10 and 75% for MK-PDMS-10.

Degradation temperatures and weight losses for all the studied systems are reported in Table 3.

### 3.2. X-ray Diffraction Characterization

**Figure 2:**
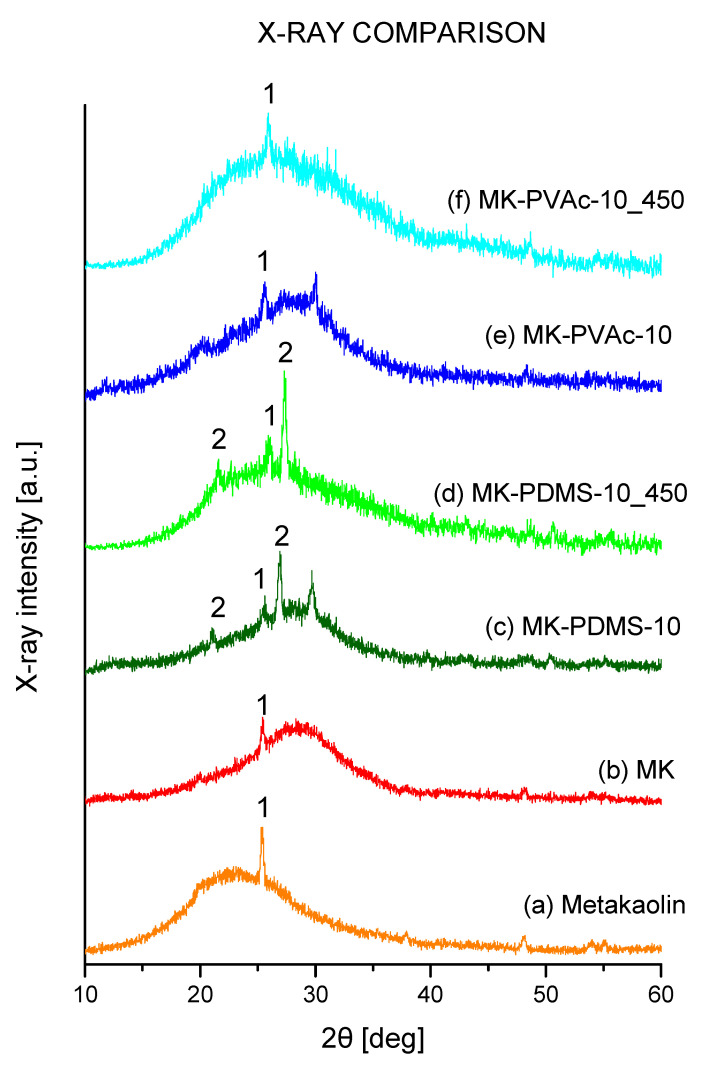
X-ray diffraction patterns of the samples: metakaolin (**a**), geopolymer (MK, (**b**)), organic–inorganic geopolymer sample containing 10%wt of polydimethylsiloxane as obtained (MK- PDMS-10, (**c**)) and after a 2 h thermal treatment in air at 450 for 2 h (MK-PDMS-10-_450, (**d**)); geopolymer composite containing 10%wt of PVAc as prepared (MK-PVAc-10, (**e**)) and after the sample has been treated at 450 °C for 2 h (MK-PVAc-10_450, (**f**)). The crystalline diffraction peaks at 2θ = 21.1°, 25.4° and 26.9° are attributable to anatase (TiO_2_, peaks marked with “1”) and quartz (SiO_2_, peaks marked with “2”) crystalline phases.

The X-ray diffraction patterns of the samples are reported in Figure 2.

The diffraction pattern of the metakaolin sample (Figure 2a, metakaolin) is characterized by a broad amorphous halo centered at 23° with only a crystalline peak at 25.4°, indicating the presence of small amounts of anatase. On the other hand, the X-ray diffraction pattern of the neat geopolymer sample (Figure 2b, MK) shows an amorphous halo (with a maximum at 2θ ≈ 29°) linked to the formation of a hydrate disordered network of Si-O-Al bonds, characteristic of geopolymerization [16,17,26]. Again, also in the geopolymer MK sample (Figure 2), the only appreciable crystalline peak at 2θ = 25.4° is reminiscent of the presence of a low amount of TiO_2_ in the starting metakaolin.

Similarly, the diffraction patterns of both organic–inorganic composite samples show an amorphous halo at the 2θ ≈ 29° with the presence of a very minor quartz phase in MK- PDMS-10. Performing an annealing treatment at 450 °C for 2 h, it is possible to note the center of the amorphous halo shifts to lower 2θ angles, of which maximum is now centered around 25° of 2θ (MK-PDMS-10_450). This is due to the almost total removal of the water molecules that hydrate the amorphous phase of the geopolymer sample. At the same time, the sample with PVAc shows the presence of just one phase (the anatase from starting metakaolin) and shows a similar variation of the amorphous halo with the shift of the maximum at 2θ value near to 25° (MK-PVAc-10_450, curve f). 

### 3.3. Morphological Characterization

**Figure 3:**
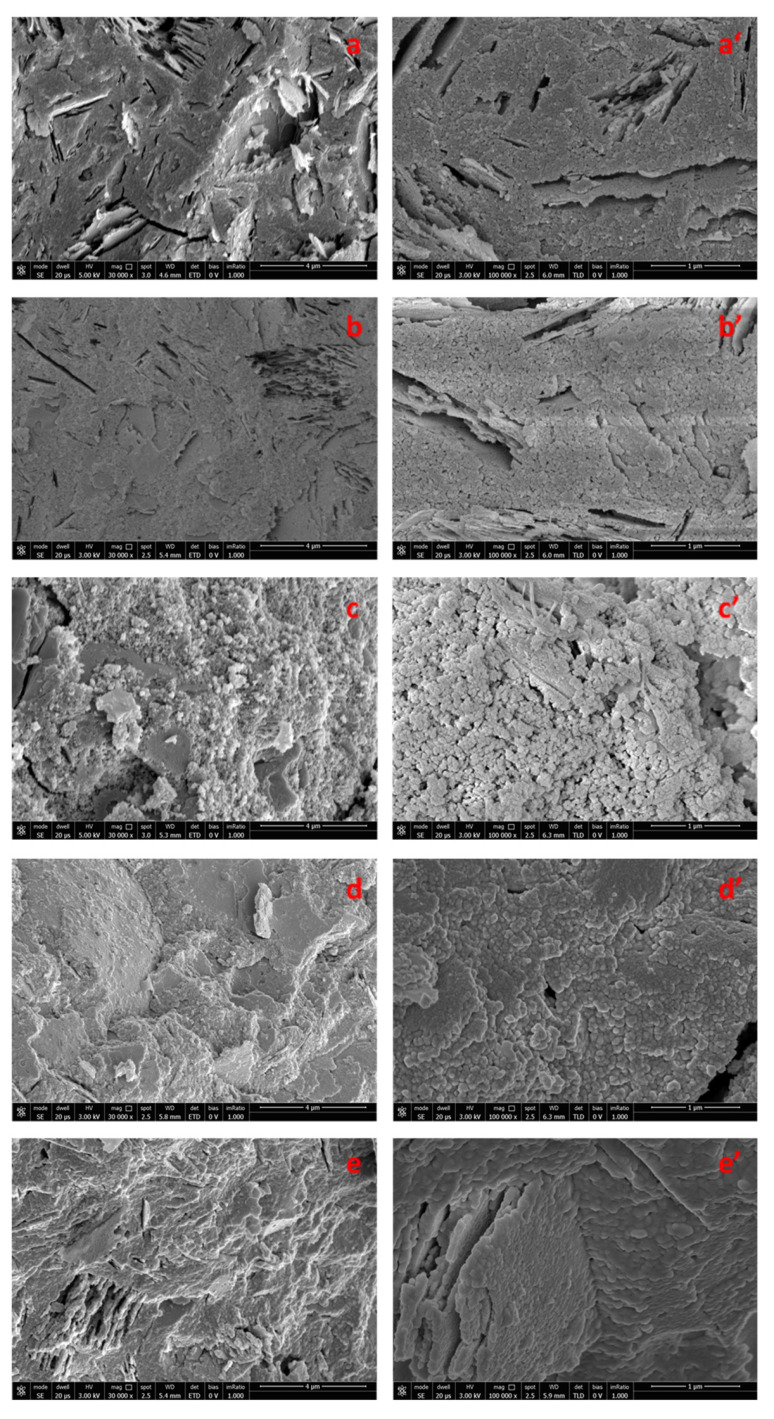
Scanning electron microscope (SEM) micrographs at 30,000× and 100,000× magnifications of freshly obtained fractured surfaces of: neat geopolymer MK (**a**,**a’**), MK-PVAc-5 (**b**,**b’**), MK- PVAc- 10 (**c**,**c’**), MK-PDMS-5 (**d**,**d’**), and MK-PDMS-10 (**e**,**e’**).

SEM micrographs of the freshly obtained fractured surfaces of the geopolymer samples are reported in Figure 3. The neat geopolymer (Figure 3a,a’) shows a homogeneous amorphous structure, where it is possible to recognize the presence of several voids reminiscent of tabular aggregates of metakaolin, visible also in high magnification images [35,36]. This structure indicates that the geopolymerization process has been successfully carried out [36].

As far as organic–inorganic samples containing PVAc (Figure 3b,b’,c,c’), a compact and homogeneous morphology is clearly observed, with a reduced number of voids compared to the neat geopolymer sample. In particular, from the examination of images 3c,c’ referring to samples MK-PVAc-10, an apparently floury structure is noted, characterized by the presence of a small quantity of unreacted material, which appears in the form of approximately lamellar areas. In this sample, which contains about 10% by weight of PVAc (and similarly also in the sample with 5% by weight of PVAc), no apparent phase separation is observed between the organic and inorganic phases. The structure, in fact, even at high magnifications (Figure 3c’), appears to be constituted by the aggregation of spherical domains deriving from the gelation reaction of the geopolymer. No evident PVAc polymer domains are observed. It is worth noticing that in the alkaline environment, in which the geopolymerization reaction has been carried out, PVAc can undergo hydrolysis to a mixed polymer containing both hydroxyl and acetyl groups [37]. The high water solubility of the partially hydrolyzed PVAc could explain the absence of domains of the organic polymer. 

By way of comparison, in Figure 3d,d’,e,e’, the SEM micrographs of the fresh fracture surfaces of the hybrid samples containing silicone (at 5% by weight, sample MK- PDMS- 5, Figure 3d,d’, and al 10% by weight, sample MK-PDMS-10, Figure 3e,e’). The morphological investigation, even at very high magnifications, also reveals a compact structure without phase separation.

**Figure 4:**
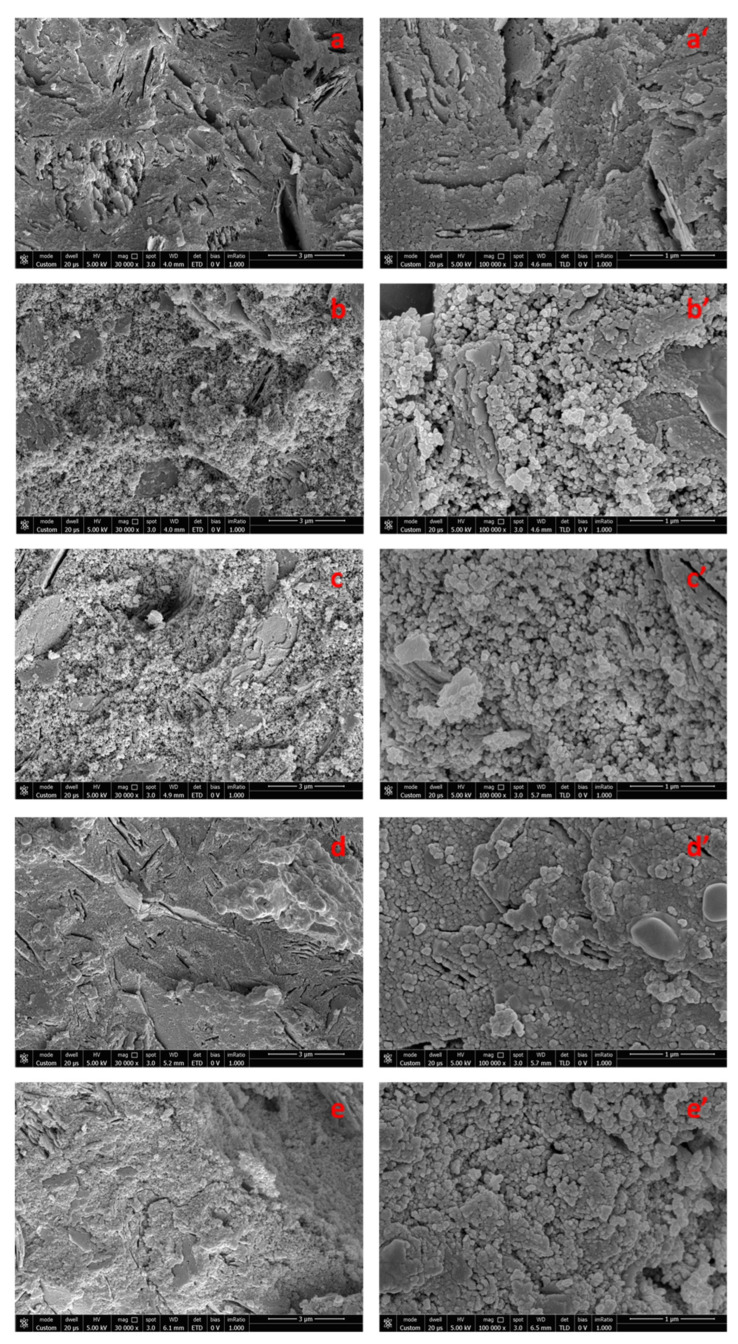
Scanning electron microscope (SEM) micrographs at 30,000× and 100,000× magnifications of freshly obtained fractured surfaces of: neat geopolymer MK (**a**,**a’**), MK-PVAc-5 (**b**,**b’**), MK- PVAc- 10 (**c**,**c’**), MK-PDMS-5 (**d**,**d’**), and MK-PDMS-10 (**e**,**e’**) after thermal treatment at 450 °C for 2 h in air.

To highlight the presence of any domains of organic nature dispersed within the geopolymeric matrix, SEM images were also recorded of the same samples after a heat treatment at 450 °C for two hours in air (Figure 4) and at 600 °C for 12 h (images not shown). These additional thermal treatments were aimed to remove the organic moieties from the samples, allowing a better understanding of the resultant microstructure. For these annealing treatments, the samples of organic–inorganic composites with a similar composition demonstrated well-defined microspheres of the organic phase that could be removed upon thermal treatments in air [26,29]. Meanwhile, the morphology of the new proposed samples subjected to heat treatment is not significantly changed compared to that of the non-heat-treated samples. These data would suggest the absence of detectable phase separation in MK-PVAc samples, for which it is possible to recognize only one phase, even at a nanometric level scale.

As a final remark, in the case of MK-PVAc samples (Figure 3b,b’,c,c’ and Figure 4b,b’,c,c’), the observed morphology is different to that of the neat geopolymer (Figure 3a,a’ and Figure 4a,a’), since it appears to be less compact, characterized by the presence of nodules with an average diameter of about 40–50 nm surrounded by a diffuse porosity. The opposite effect is detectable in the case of the MK-PDMS samples (Figure 3d,d’,e,e’ and Figure 4d,d’,e,e’), for which a more compact nodular morphology with respect to the neat MK geopolymer samples is apparent. These differences could allow for rationalizing the different mechanical behaviour of the studied specimens, as discussed in the next section.

### 3.4. Physical and Mechanical Properties

Table 4 shows the physical properties of the investigated samples (i.e., bulk density and water absorption). MK sample shows the highest value of density among the investigated samples. The addition of the polymeric phase (from 5 to 10 wt.%) slightly, but progressively, reduces the material density. With regards to the water absorption of the MK neat samples and MK-PVAc series, no remarkable differences are found. Data concerning the MK-PDMS samples are affected by the hydrophobicity of polydimethylsiloxane, which hinders contact with water. Consequently, the noticeable decrease in water absorption cannot be compared with the other results. 

**Figure 5:**
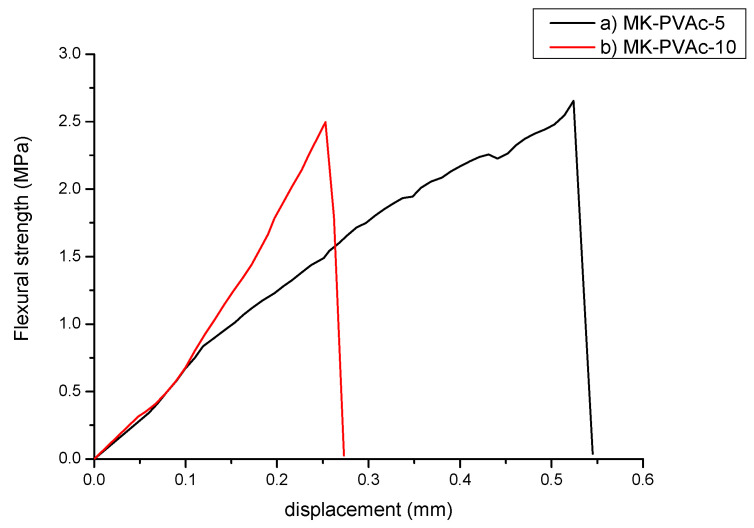
Flexural strength vs. displacement for relevant samples: (**a**) MK-PVAc-5; (**b**) MK- PVAc- 10.

Table 5 shows the mechanical properties of the investigated samples: dynamic elastic modulus, flexural strength, and compressive strength. MK shows the highest values of E. The addition of the polymeric phase, both PVAc and PDMS, decreases the dynamic elastic modulus of samples, as could be expected. The presence of PVAc significantly increases (up to 30%) the value of flexural strength. In particular, Figure 5 compares the plots reporting the flexural strength vs. the deflection of MK-PVAc samples with 5 and 10% in the weight of PVAc, respectively. A representative sample for each of the two series is reported. A visible toughness increase takes place when 5% in the weight of PVAc is present, which is less detectable in all the other series. The addition of a higher amount of PVAc does not seem to positively modify the properties of the composite materials. 

**Figure 6:**
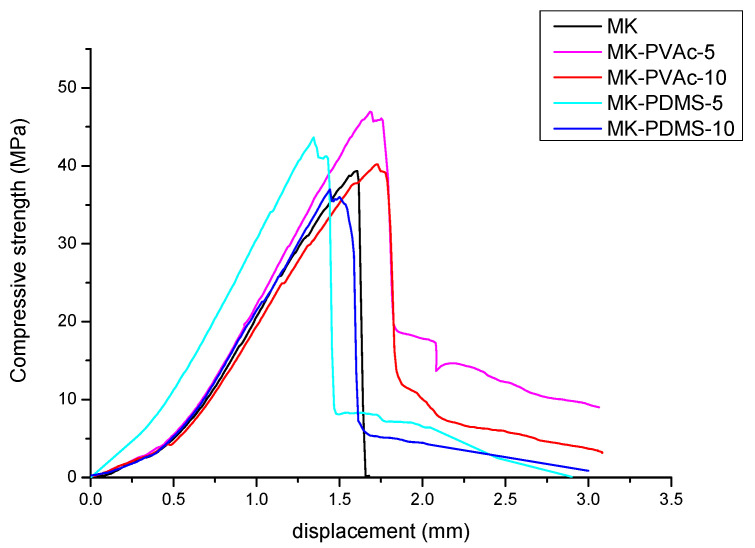
Compressive strength vs. displacement for samples: (black) MK; (pink) MK-PVAc-5; (red) MK- PVAc-10; (sky blue) MK-PDMS-5; (blue) MK-PDMS-10.

As far as compressive strength, all materials demonstrate outstanding values (Table 5) and no remarkable differences can be detected among the different compositions. Figure 6 shows the plots reporting the compressive strength vs. the deflection of a representative sample of each series. The toughening effect of the PDMS and PVAc addition is reflected by the increase in the area beneath the plot (from 15 to 40%), mainly in the second part of the curve. The highest amount of absorbed energy is relevant to all samples in which the 5 wt.% addition is present and is more evident for MK-PVAc samples (Figure 6). The demonstrated results could be further highlighted by impact strength tests that are in progress.

### 3.5. Applications in the Field of the Creative Industry and Cultural Heritage

The geopolymer composites have been subjected to preliminary tests to evaluate their potentialities as sacrificial and fixing materials for technical–artistic and cultural heritage applications. 

**Figure 7:**
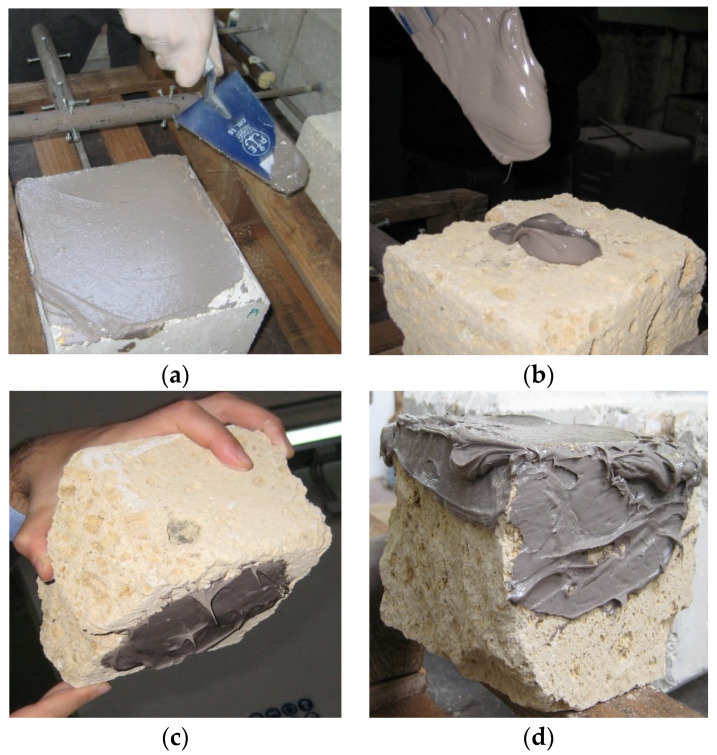
Preliminary adhesion test of MK-PVAc-10 soon after its preparation on different substrates: (**a**) cement; (**b**–**d**) tuff. A very good thixotropic behavior and very high workability are apparent.

Experimental adhesion tests conducted with MK-PVAc-10 mixture soon after its preparation have highlighted that the addition of the organic phase to the aluminosilicate matrix allows for obtaining a thixotropic behavior and high workability, which makes it easy to model and spread on different substrates that need repair and fixing interventions (Figure 7). 

**Figure 8:**
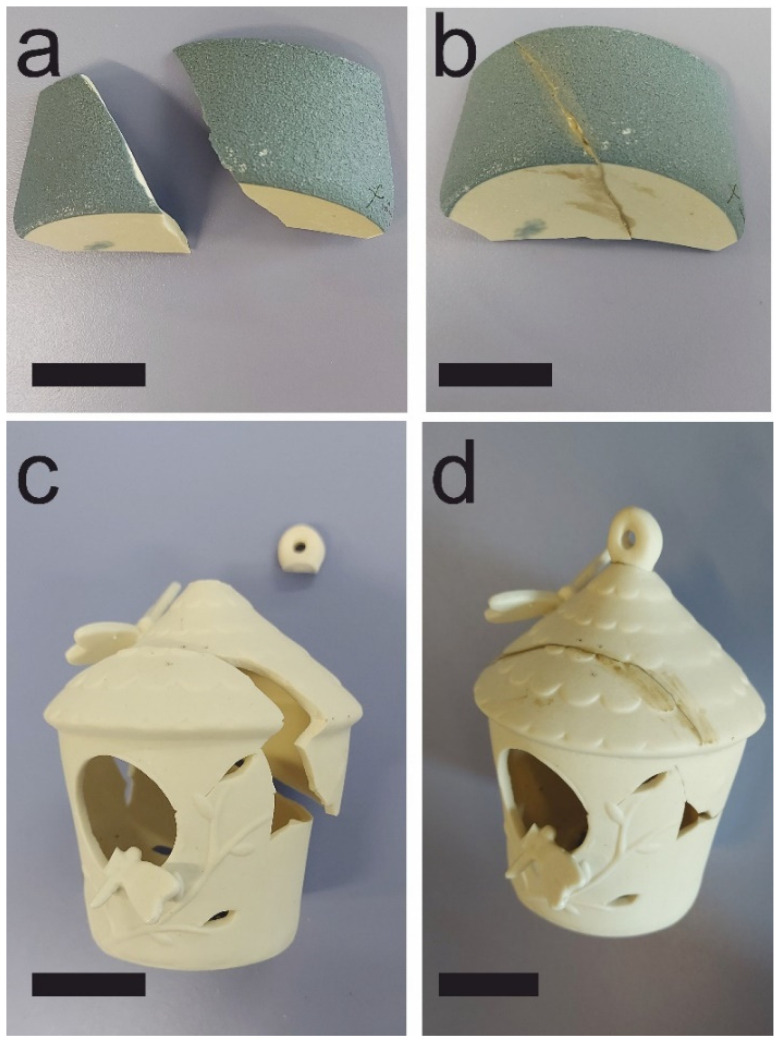
Porcelain stoneware and pottery artworks (**a**–**c**) were restored (respectively in (**b**–**d**) picture) and repaired by using the geopolymer-based material MK-PVAc-10 prepared in this work (black scale bar indicates 5 cm).

In addition, repair work tests were carried out on porcelain and stoneware artefacts, as shown in Figure 8. The artefacts were restored and repaired by using the MK-PVAc-10 pastes as fixing materials. A very good adhesion in each of the examined cases has been observed. As reported in previous works [17], the organic phase makes an important contribution to the workability of the geopolymer blend by increasing the viscosity if compared to a neat geopolymer. This behavior makes it possible to obtain a thixotropic mixture that avoids the dripping phenomena, which would make any repair and restoration work difficult. Moreover, no shrinkage or micro-cracking phenomena have been evidenced even months after the repair operation, pointing to the possibility of using this kind of binder as an effective long-term joining or fixing paste.

It is worth pointing out that the geopolymer composite materials developed in this work are easily recognizable with respect to the support on which they are used due to their organic–inorganic hybrid nature, which can be easily highlighted, for example, by IR spectroscopy. In addition, in accordance with the provisions of the restoration protocols, precisely because of this easy recognition, these materials are also easily removable from the support on which they were used. 

The good adhesion between MK-PVAc-10 composite with a porcelain substrate was confirmed by carrying out the SEM characterization of the interfacial transition zone between the geopolymer composite, used as fixing material, and a pottery fragment.

**Figure 9:**
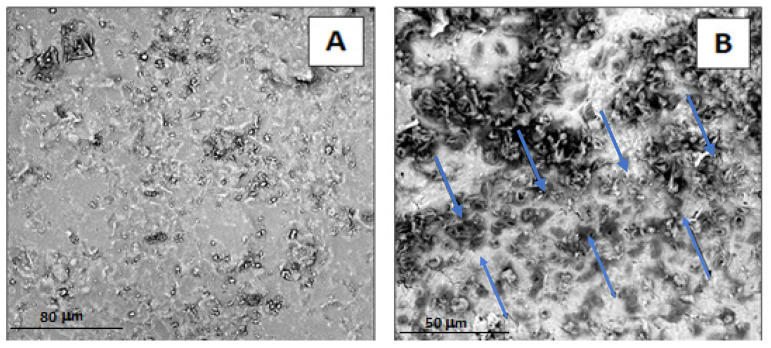
Scanning electron microscope (SEM) micrographs of (**A**) ceramic substrate and (**B**) interfacial transition zone (indicated by the blue arrows) between the ceramic substrate (upper part of the figure) and MK-PVAc-10 geopolymer composite (low part of the figure).

SEM micrographs (Figure 9) show the presence of a very strict adhesion between the geopolymer composite and ceramic phase up to the micrometric level (see Figure 9B). Thus, it is possible to observe the formation of a continuous phase and the complete absence of microfractures and voids, that, if present, could weaken the structure and make restoration work ineffective over time.

**Figure 10:**
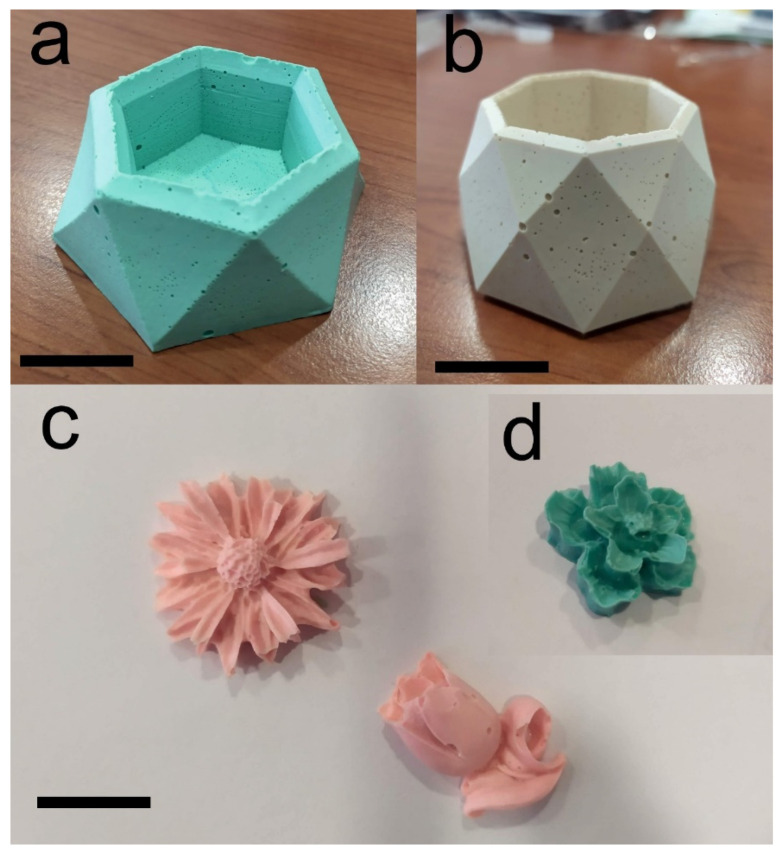
Examples of painted artefacts created with the geopolymer composite materials and presented in this paper. The artistic products were created by adding pigments to geopolymer paste and a simple pouring procedure. In (**a**,**b**) the scale bar is 4 cm, while in (**c**,**d**) the scale bar is 2 cm.

Finally, it is worth pointing out that the geopolymer composites developed and characterized in this work can be easily colored by simply adding water and/or oil-based pigments into the slurry and/or through post-painting operations with cold painting. In fact, the presence of the polymeric phase would appear to stabilize the pigments, preventing phase separation and blending phenomena, which would consequently be unsightly to artistic artefacts. As an example, Figure 10 reports the different kinds of artefacts realized with the MK-PVAc-10 composite, with the addition of water-based pigments.

It is worth mentioning that a wide range of both organic and inorganic consolidants useful to repair different types of materials are already available on the market. They can be divided into three main classes: (i) organic products (e.g., alkoxysilanes) [38,39]; (ii) organic–inorganic mixtures [40,41] and (iii) purely inorganic products, usually cementitious and apatitic materials [42]. The use of organic polymeric materials provides good hydrophobicity and protection while contributing little to improving mechanical properties. Therefore, polymers additivated with metal oxides (e.g., titanium dioxide) are used to achieve improvements in the mechanical component and have antimicrobial functionality [43]. In contrast, inorganic materials can make an important structural contribution [44], but they are often materials that do not protect against moisture and give saline releasing phenomena. In all cases, the main issue lies in the lack of reversibility of the restoration, as the material used is not easily eliminated from the support on which it was used. On the contrary, the geopolymer composite materials studied in this work overcome all these limits since, in addition to presenting good mechanical properties and easy workability, they are at the same easily recognizable by spectroscopy thus meeting the fundamental criteria of reversibility to the restoration.

As a final consideration, it is worth pointing out that the new materials presented in this work should be tested in the outdoor durability (simulating the external environmental conditions) to evaluate their stability over time, even in high humidity or rainfall, to be used as restoration and repair materials for outdoor monuments or buildings. Currently, a limitation in the application of these systems could be their use limited to in indoor or controlled environments.

## 4. Conclusions

The present research describes the preparation of new geopolymer-based composite materials obtained by adding up to 10% by weight of polyvinyl acetate (PVAc) to the geopolymer mixture to obtain cheap and sustainable materials for the application in the field of art and design. Their morphological, physical, and mechanical characterizations were carried out and compared with analogous polydimethylsiloxane–geopolymer hybrid materials. 

Particularly, geopolymer-PVAc composites prepared and described in this paper demonstrate improved flexural strength and a relevant toughening effect if compared to the neat geopolymer. In particular, the geopolymer-PVAc composite materials have demonstrated better physical and mechanical performances if compared to the neat geopolymer and hybrid materials with polydimethylsiloxane: lower density (up to 15%), an improved flexural strength (up to 30%), similar water absorption, and a relevant toughening effect (up to 40%). These features point out the possible applicability of such systems in the field of building, restoration, conservation of artworks, and the creation of products for art and design. In fact, the excellent mechanical properties and low water absorption make the proposed materials useful in repair and fixing, and make repair and fixing works stable and reliable in the long term, even outdoors. The composite materials developed in this work succeed in meeting all the criteria required of materials used in the field of restoration and repair, unlike those currently in use, which, taken individually, fail to do so. Moreover, the good adhesion of these new geopolymer composites to different substrates (cement, tuff, ceramic, and porcelain stoneware) and a good thixotropicity (the presence of organic phase increases the viscosity of the composite pastes with respect to neat geopolymer) ensure good adhesion with decorative and structural systems, also avoiding unpleasant dripping phenomena and, at the same time, demonstrating the criteria for reversibility. 

As a final remark, it is worth pointing out that the development and use of innovative binding materials with a lower environmental footprint, if compared to traditional cementitious materials, can provide an important contribution in the area of art and design and cultural heritage (and, in particular, in the field of restoration and rehabilitation of artistic heritage), by contributing to the further reduction of their overall environmental footprint. Under this point of view, the environmental impacts related to the production of the new materials proposed and compared with the organic, inorganic, and mixed materials currently used in these sectors will be examined in future works.

## Figures and Tables

**Table 1:** Chemical composition (weight %) of the used metakaolin (BASF MetaMax^®^) and sodium silicate solution adapted from [17], MDPI 2022.

Compound	Metakaolin	Sodium Silicate
SiO_2_	52.2	27.40
Al_2_O_3_	45.1	-
Na_2_O	0.22	8.15
K_2_O	0.15	-
TiO_2_	1.75	-
Fe_2_O_3_	0.42	-
CaO	0.04	-
MgO	0.04	-
P_2_O_5_	0.08	-
H_2_O	-	64.42

**Table 2:** Mix design of the geopolymeric samples studied in this paper.

Materials (Weight %)	MK	MK-PVAc-5	MK-PVAc-10	MK-PDMS-5	MK-PDMS-10
Metakaolin	37.5	35.7	33.7	35.7	33.7
NaOH	7.2	6.8	6.5	6.8	6.5
Sodium silicate	55.3	52.5	49.8	52.5	49.8
Polyvinyl acetate	-	5.0	10.0	-	-
Polydimethylsiloxane oligomers	-	-	-	5.0	10

**Table 3:** Thermal properties of the neat geopolymer MK, MK-PVAc-10, and MK-PDMS-10 samples.

Samples	Wt_s_ (°C) ^1^	Wt_e_ (°C) ^2^	R (Weight %) ^3^
MK	30	300	84
MK-PVAc-10	30	720	78
MK-PDMS-10	30	500	75

^1^ Wt_s_: weight loss starting temperature (°C); ^2^ Wt_e_: weight loss ending temperature (°C); ^3^ R: residual at 800 °C (weight %).

**Table 4:** Physical properties of the investigated samples: bulk density, water absorption, and total open porosity.

Sample	Bulk Density (g/cm^3^)	Water Absorption (%)
MK	1.35 ± 0.03	33.8 ± 0.4
MK-PVAc-5	1.23 ± 0.01	31.9 ± 0.1
MK-PVAc-10	1.16 ± 0.01	34.6 ± 0.1
MK-PDMS-5	1.32 ± 0.01	8.6 ± 0.1
MK-PDMS-10	1.29 ± 0.01	6.5 ± 0.1

**Table 5:** Mechanical properties of the investigated samples: dynamic elastic modulus (E), flexural strength (σ_f_), and compressive strength (σ_c_).

Sample	E (GPa)	σ_f_ (MPa)	σ_c_ (MPa)
MK	7.5 ± 0.3	2 ± 1	40 ± 5
MK-PVAc-5	6.2 ± 0.8	2.7 ± 0.2	42 ± 5
MK-PVAc-10	5.1 ± 0.2	2.6 ± 0.2	38 ± 2
MK-PDMS-5	5.8 ± 0.2	1.1 ± 0.4	42 ± 2
MK-PDMS-10	4.9 ± 0.2	0.7 ± 0.1	37 ± 2

## Data Availability

The authors declare the availability of the data reported in this paper.
